# The history of neuromyelitis optica

**DOI:** 10.1186/1742-2094-10-8

**Published:** 2013-01-15

**Authors:** Sven Jarius, Brigitte Wildemann

**Affiliations:** 1 Division of Molecular Neuroimmunology, Department of Neurology, University of Heidelberg, Im Neuenheimer Feld 450, 69120, Heidelberg, Germany

**Keywords:** Neuromyelitis optica, Devic’s syndrome, Devic’s disease, Myelitis, Optic neuritis, Multiple sclerosis, Nomenclature, Diagnostic criteria, History of neurology

## Abstract

The discovery of a novel serum autoantibody (termed NMO-IgG or AQP4-Ab) in a subset of patients in 2004 has revived interest in neuromyelitis optica (NMO). While the history of classical multiple sclerosis has been extensively studied, only little is known about the history of NMO. In the present article, we provide a comprehensive review of the early history of this rare but intriguing syndrome. We trace the origins of the concept of NMO in the 19th century medical literature and follow its evolution throughout the 20th and into the 21st century. Finally, we discuss recent proposals to revise the concept of NMO and explain why there is indeed a need for a more systematic and descriptive nomenclature.

## Introduction

Neuromyelitis optica (NMO) is a rare condition, characterized by myelitis and optic neuritis, which shares a number of clinical and radiological features with multiple sclerosis (MS) [1-3]. The groundbreaking discovery of a novel, pathogenic autoantibody (termed NMO-IgG or AQP4-Ab) in a subset of patients by Dr Lennon and colleagues in 2004 [4,5] has led to a tremendous increase in interest in NMO. NMO-IgG/AQP4 antibody-positive NMO is now considered a disease entity in its own right rather than a subtype of MS.

While the history of classical MS has been studied extensively, only little is known about the history of NMO. In the present article, we comprehensively review the early history of NMO. We trace the first accounts of this peculiar term in the 19th century French-, English-, and German-language literature and follow its definition’s meandering evolution throughout the 20th and into the 21st century. Finally, we will discuss recent proposals to re-define or substitute the term and explain why there is indeed a need for a more systematic and descriptive nomenclature.

### Eugène Devic and Fernand Gault

**Figure 1:**
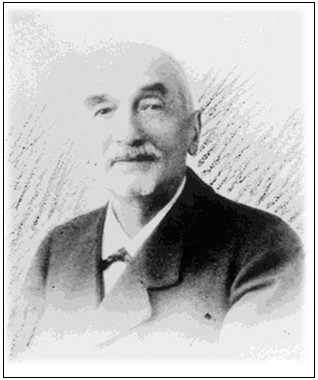
Eugène Devic (1858–1930).

**Figure 2:**
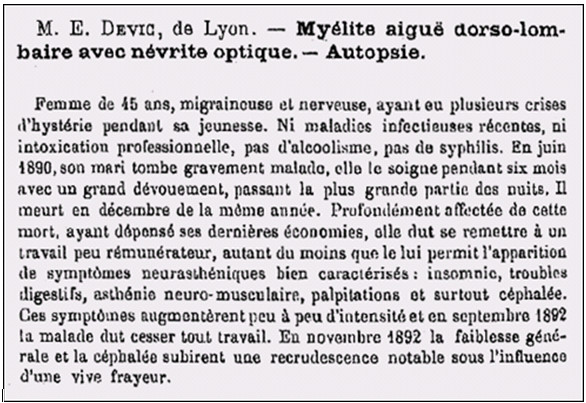
**Heading and first paragraph of Eugène Devic’s famous abstract for the *Congrès Français de Médecine *in Lyon in 1894.**

‘Neuromyelitis optica acuta’ and the more rarely used English equivalent ‘acute optic neuromyelitis’ are both translations of the French term ‘neuro-myélite optique aiguë’, which was first used by Eugène Devic (1858–1930) in a paper communicated on the occasion of the *Congrès Français de Médecine* in Lyon in 1894 (Figures 1 and 2). Devic intended the term to denote a novel syndrome characterized by acute myelitis and optic neuritis: ‘Ces seize cas de myélite aiguë accompagnés de névrite optique sont suffisants pour légitimer la création d’un type clinique, ou plutôt d’un syndrome auquel on pourrait donner le nom de *neuro*-*myélite optique*’ [6].

**Figure 3:**
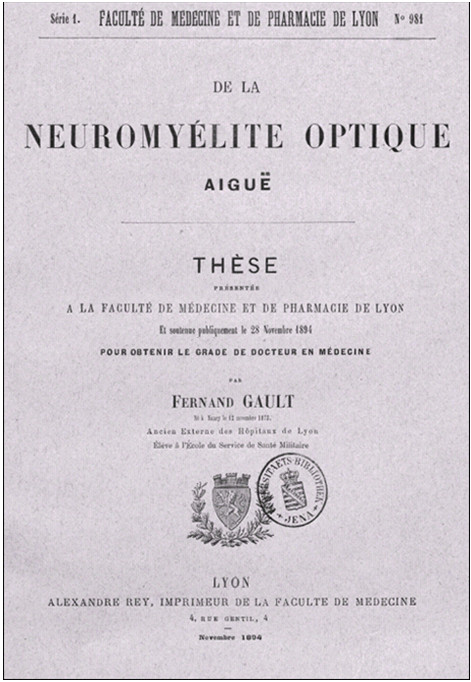
**Title page of Fernand Gault’s doctoral thesis *De la neuromyélite optique aiguë *(Lyon, November 1894).**

The same year, Devic’s student Fernand Gault (1873–1936) published his doctoral thesis, entitled *De la neuro*-*myélite optique aiguë* (Figure 3), which consisted of a review of the previous medical literature and a clinicopathological analysis of Devic’s case [7]. (The fact that the congress proceedings with Devic’s abstract appeared only in 1895, i.e. after Gault’s thesis, has given rise to some confusion in the literature. However, Devic gave his presentation on Friday, 26th October 1894; by contrast, Gault’s thesis was printed only in November 1894 according to the imprint, and its dedication is dated ‘20 novembre 1894’.)

We will never know with absolute certainty whether it was Devic or Gault who originally invented the term; however, the following lines from Gault’s thesis strongly suggest it was indeed Devic: ‘Bien que l’axiome « Il n’y a pas de maladies, il n’y a que des malades » soit toujours vrai, il est certain cependant que, quand on parvient à réunir en un syndrome un ensemble constant de symptômes, il est certain, dis-je, que l’on peut proposer un nom servant à désigner le cas type, et sur les conseils de M. le Dr Devic, je propose celui de neuro-myélite diffuse, aiguë’ [7].

There is some evidence that the term ‘neuro-myélite’ was chosen by Devic and Gault in analogy with ‘neuro-cérébrite’, a term associated with the name of August Pierret. Pierret, a pupil of Charcot and at that time professor at the *Clinique des maladies mentales* at Lyon, was president of the examination board committee responsible for Gault’s thesis. Moreover, Gault’s thesis was dedicated to Pierret, and its very last sentence reads: ‘Ce syndrome relève probablement dans un certain nombre de cas de l’infection. C’est un exemple nouveau d’un processus infectieux, frappant à la fois deux points éloignés du système nerveux sans qu’il y ait de lésions anatomiques quelconques reliant ces deux foyers. A ce titre, ce syndrome mérite d’être rapproché de la neuro-cérébrite de M. le professeur Pierret’ [7].

(In 1935, the Berlin neurologist Erwin Stengel would make an attempt to resuscitate Pierret’s term in a paper on a series of patients with neuritis cranialis and accompanying brainstem encephalitis, in which he proposed to refer to such cases by the term ‘neuro-encephalitis’. Interestingly, one of his patients had optic neuritis, and Stengel wrote: ‘Der Fall, bei dem auch eine Neuritis optica bestand, könnte in Analogie zur Bezeichnung Neuromyelitis optica als *Neuroencephalitis optica* [italics ours] bezeichnet werden’ [8]. Recent studies have shown that brainstem involvement is quite common in patients with AQP4 autoimmunity, and cases of AQP4 antibody-positive ‘neuroencephalitis optica’ without concomitant myelitis indeed occur [1,9]. —A very early account of possible ‘neuroencephalitis optica’ is to be found in the in the second, enlarged edition (1829) of John Abercrombie’s (1780–1844) *Pathological and Practical Researches on Diseases of the Brain and Spinal Cord*, a case of intractable vomiting, relapsing visual loss, and spinal pain [10,11]; intractable vomiting and hiccups are typical manifestations of medulla oblongata involvement in aquaporin-4 antibody-positive NMO and often herald the onset of myelitis [12,13]).

While several articles on Devic’s life have been published, only little is known about Gault’s biography. According to Politzer’s monumental *Geschichte der Ohrenheilkunde*, Gault worked as an army doctor following his graduation from the Lyon faculty in 1894, but soon became professor of otorhinolaryngology at the Medical School in Dijon (in 1905) and head of the ENT department there in 1911 [14]. We traced more than 20 publications authored or co-authored by Gault; however most of them were dedicated to ENT topics. It seems that Gault never again published on NMO. He died in 1936, just six years after Devic.

### NMO before Devic and Gault

As recently shown by us [10,15-17], Devic and Gault in their reviews overlooked some early cases of possible NMO, probably owing to the restricted bibliographic resources of the time (both Devic’s and Gault’s lists of references were mainly based on that of an earlier German review by the Dresden-based ophthalmologist Fritz Schanz [18], as conceded by Devic in reference [19]). In 1844, i.e. 26 years prior to the first case referenced by Devic and Gault, the Genoese physician Giovanni Battista Pescetto (1806–1884) had reported on a 42-year-old man who simultaneously developed acute amaurosis and cervical myelitis, with complete recovery following extensive bloodletting [10]; in 1850, the British physician Christopher Mercer Durrant (1814–1901) had described a case of tetraparesis and (partly reversible) bilateral amaurosis in the precursor of the *British Medical Journal*[20]; and in 1862 the British neuroanatomist, neuropathologist, and neurologist Jacob Augustus Lockhart Clarke (1817–1880), known to many as the eponym of Clarke’s column, the posterior thoracic nucleus, had reported the case of a 17-year-old girl with bilateral optic neuritis and longitudinally extensive transverse myelitis in *The Lancet*[15]. Finally, a report by Antoine Portal (1742–1832), first physician to Louis XVIII and founding and lifelong president of the Académie Nationale de Médecine, deserves to be mentioned here: it represents the first account of visual loss in a patient with spinal cord inflammation but no brain pathology in the Western literature known so far [16]. However, none of these previous authors had ever used the term ‘neuromyelitis optica’ or a similar one.

### Peppo Acchioté

In 1907, Peppo Acchioté (1870–1916; the original Turkish spelling is Pe(p)po Akşiyote or Akşiyoti (Kirbaş, 2003)), a physician from Constantinople, ‘spécialiste pour les maladies nerveuses et sur l’électrothérapie’ [21], proposed for the first time - in a paper communicated by no less a figure than Joseph Babinski (1857–1932) on 4th July 1907 on the occasion of a session of the *Société de Neurologie de Paris* - to make Devic the eponym of NMO: ‘L’association de névrite optique avec une myélite diffuse constitue l’affection denommée par M. Devic, de Lyon, *neuromyélite optique aiguë* et qu’il serait plus juste, à mon avis, de designer sous le nom de *maladie de Devic*’ [22].

In this paper, Acchioté also reported on a case of his own, a 25-year-old woman with bilateral optic neuritis, paraparesis, and sensory and sphincter disturbances.

In choosing the term ‘*maladie*’, Acchioté deviated from Devic’s original definition of NMO as a ‘*syndrome*’ or ‘*type clinique*’ [6]. This deviation has consequences to our day, as we will discuss below. It should not go unmentioned, however, that Acchioté’s choice of the word ‘maladie’ has in fact some foundation in Gault’s thesis. While Devic originally described NMO as a ‘syndrome’ or ‘type clinique’, Gault was more ambiguous. While he defined ‘NMO’, following Devic, as a ‘syndrome’ or ‘complexus symptomatique’ in the final conclusion of his thesis - characterized by bilateral optic neuritis (simultaneously or alternately), usually resulting in complete yet mostly transient amaurosis (with possible full functional recovery), and diffuse or localized acute myelitis, with the latter mostly following the optic nerve affliction - he wrote in the introduction to his thesis: ‘Le syndrome dont nous voulons parler constitue cependant une *entité morbide* [italics ours] bien distincte et ayant droit de cité dans le cadre nosologique’ [7]. Elsewhere in his paper, Acchioté used the more neutral French term ‘affection’ [22].

**Figure 4:**
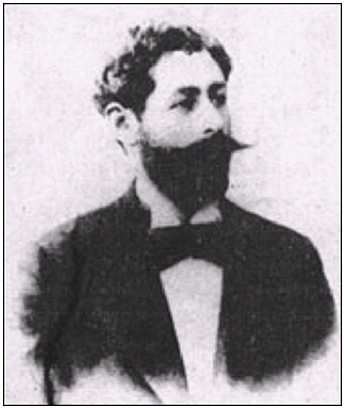
Peppo Acchioté (1870–1916).

Little is known about Acchioté’s life. According to Dursun Kirbaş’s *History of Turkish Neurology* (*Türkiye Nöroloji Tarihçesi*), Acchioté (Figure 4) studied in Paris [23]. We could identify a proceeding that lists him as a visiting physician at the *Service des enfants idiots*, *épileptiques et arriérés* at the Bicêtre in 1903 [21]. Later, he was a member of the teaching staff at the Istanbul Civilian Medical School and, at least from 1910 on, assistant professor at the Neurology Department of the Haydarpaşa Medical Faculty, as deputy to Raşit Tahsin, a pupil of Kraepelin, Binswanger, and Mendel, and one of the pioneers in neurology in Turkey [24].

Given that numerous case reports and at least two reviews [11,19] on the association of myelitis and amaurosis were published prior to Devic and Gault, Acchioté’s proposal may be considered another example of what Robert Merton (rather playfully) once called the *palimpsestic* (or *anatopic*) syndrome, which is not so rare a phenomenon in the history of medicine: ‘the altogether innocent transmitter becomes identified as the originator of the idea when his merit lies only in having kept it alive (…) or perhaps in having put it to new or instructive use’ [20]. That said, not mentioning Devic and Gault’s unique contribution would mean committing what Merton called the ‘sin of adumbrationism’ [20]: While others had in fact reported on NMO before, it was they who gave it a name - and ‘[w]hat is a disease before it gets a name?’ (T. Jock Murray, *Multiple Sclerosis: The History of a Disease*, New York, 2005).

Acchioté’s proposal was soon made known to a broader audience by the reprinting of his paper in the *Revue neurologique*[25] and by a short summary that appeared later the same month in the *Annales d’oculistique*[26].

Some authors have proposed to revise Acchioté’s eponymous designation to acknowledge also Gault’s important contribution to the history of NMO [27,28].

### ‘NMO’: early accounts in the non-French literature

Devic never used the neoclassical adaption ‘neuromyelitis optica’ widely preferred today. The earliest use of that version we could trace is found in a 1904 German-language review by the Austrian psychiatrist Erwin Stransky [29], a pioneer in research on schizophrenia (for a brief sketch of Stransky’s disturbing biography see ref. [30]). In this article, Stransky summarized and discussed a report by the French pathologist and neurologist Édouard Brissaud, a pupil of Charcot and Lasègue, about a 16-year-old boy with NMO, which had appeared in the *Revue Neurologique* earlier the same year [31].

The first two uses of the English-language term ‘acute optic neuromyelitis’, and at the same time the first account of a variant of ‘NMO’ in any language other than French we could find, were in a September 1903 issue of the *British Medical Journal*[32], in an anonymous review of Weill and Gallavardin’s case in the *Lyon Medicale*[33] (an exact reprint of that review appeared in the December 1903 issue of *The Ophthalmoscope*[34]), and in a short review by Hugh T. Patrick, Professor of Neurology at Northwestern University, of the same French report in the 10th volume of the 1904 edition of the *Practical Medicine Series of Year Books*, which appeared the same month [35]. Also in 1904, Gowers’ authoritative textbook on the ophthalmoscopic signs of neurological diseases appeared in a revised edition [36]. However, Gowers, who was well aware of the still relatively rare reports on the coincidence of optic neuritis and acute myelitis and who had taken part in the early discussions regarding the pathogenic relationship between these two afflictions, did not adopt the term ‘neuromyelitis’ in this or, to the best of our knowledge, in any other of his writings.

While most later authors writing in English and German would use the neoclassical term (rare exceptions are Perrit [37] and Vernant *et al*. [38]), local spellings (‘*neuromielite ottica*’ and ‘*neuromielitis óptica*’, respectively) were used in some of the early Italian- [39,40] and Spanish-language [41] publications on NMO, and Devic’s original version has remained prevalent in the French-language literature to this day.

### Alternative denominations

In his communication, Devic proposed ‘neuroptico-myélite’ as an alternative denomination. However, this term has not been widely adopted by neurologists (rare exceptions are to be found in Bouchut and Dechaume [42], and Euzière and Bremond [43]). In his thesis, Gault once used the term ‘neuro-myélite diffuse aigüe’; which refers to ‘myélite diffuse’ but, surprisingly, makes no mention of the optic nerve. Other rare variants used in a handful of publications include ‘neuro-optic myelitis’ [44], ‘neurópticomielitis aguda’ [45], ‘neuropticomielitis’ [41], ‘opthalmoneuromyélite’ [46-48], ‘oftalmomielitis’ [41], and ‘mielitis oftálmica’ [41].

### Etymology

Evidently, ‘neuromyelitis optica’ and ‘neuropticomyelitis’ are artificial composites of myelitis, i.e. inflammation of the spinal cord, and ‘neuritis (cranialis) optica’, a problematic (strictly speaking, a neuritis cannot be ‘optical’; however, as a sort of *constructio ad sensum*, some may nevertheless consider that expression acceptable) and, accordingly, not widely used (a famous exception is Wilhelm Erb’s early description of NMO, which was entitled *Über das Zusammenvorkommen von Neuritis optica und Myelitis subacuta*[49]; see also Noyes [50]) alternative to the more correct term ‘neuritis nervi optici’, i.e. inflammation of the optic nerve. Etymologically, they are combining forms of the Ancient Greek words *νε\scale80%ῦρον*, i.e. nerve, sinew or tendon, and *μυελός*, i.e. marrow (probably a derivative of *μυών*, muscle), the post-classical Latin word *optica*, and the suffix *-itis*, which was already used in Greek to indicate disease (though not necessarily inflammation). As ‘neuropticomyelitis’ is a Greek-Latin hybrid and because ‘optic myelitis’ does not, strictly speaking, make much sense, purists might regard these terms as linguistic barbarism [51]. However, such classical compounds are common in medical terminology with its strong need for classification and, in consequence, distinguishing denominations; accordingly, the term was widely accepted and has survived to our days.

### Previous usage of the term ‘neuromyelitis’: Dunglison and Hildenbrand

It is little known that compounds of *νε\scale80%ῦρον* and *μυελός* had been in existence before Devic’s time. The 1836 edition of the prestigious *Dictionnaire de l’Académie Française* defined ‘névromyélite’ as ‘inflammation de la moelle épinière’ and, thus, as a synonym of ‘myélite’. However, we did not find evidence in the medical literature of the time that the term thus defined was widely used; instead, it seems to have led a lonely existence in that dictionary as a *hapax legomenon*. The same holds true for the (etymologically corrupt) definition of ‘neuro-myelitis’ as inflammation of the vertebra in an 1875 Italian dictionary [52]. However, the etymological explanations given above indicate that the term ‘neuromyelitis’ may also be used (and perhaps with more justification from a linguistic point of view) to refer to inflammation of the *medulla nervorum*. In fact, the word was defined in Robley Dunglison’s (then widely used) 19th century *Medical Lexicon* exactly in this sense: ‘NEUROMYELI’TIS, from *νευρον*, “a nerve”, *μυελοs*, “marrow”, and *itis*, denoting inflammation. Inflammation of the medullary matter of the nerves’. This definition is first documented in the 1848 edition of that early *Dictionary of Medical Science* (as its subtitle reads), and we found it in a number of (later) German dictionaries as well (cf. Ernst Gabler*, Lateinisch-deutsches Wörterbuch für Medicin und Naturwissenschaften*, Berlin, 1857: ‘Neuromyelitis, die Entzündung des Nervenmarks. Neuromyelos, das Nervenmark’, Wilhelm Probstmayr, *Etymologisches Wörterbuch der Veterinär-Medicin und ihrer Hilfswissenschaften*, Munich, 1863: ‘Neuromyelitis (…), die Entzündung des Nervenmarks = *Inflammatio medullae nerveae*’).

**Figure 5:**
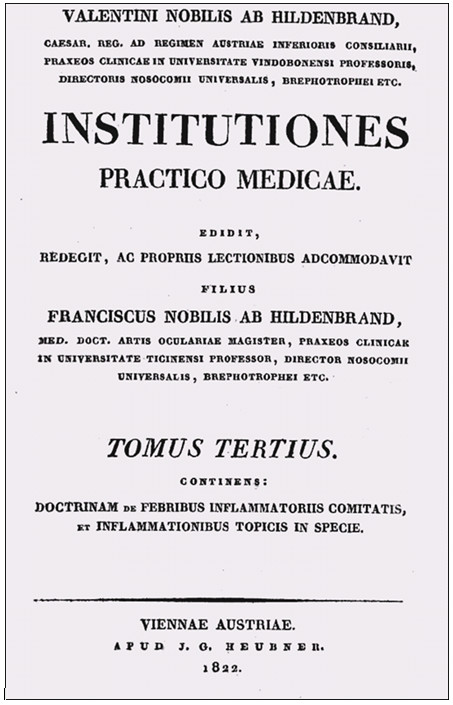
**Title page of the third volume of Johann Valentin Hildenbrand’s *Institutiones practico*-*medicae *(Vienna, 1822; published posthumously).**

One of the few accounts of the term ‘neuromyelitis’ as defined by Dunglison (or, more exactly, ‘nevromyelitis’) - and at the same time the earliest one we are aware of - in the medical literature is to be found in the third volume of Johann Valentin Hildenbrand’s famous *Institutiones practico-medicae* (published posthumously by his son in 1822) [53] (Figure 5). Hildenbrand (1763–1818) used the term to distinguish inflammation of the *pulpa nervorum* from that of the *vagina nervorum*, to which he referred as ‘nevrilemmatitis’: ‘Longe obscurius incedit phlogosis pulpae nervosae (Nevromyelitis); confunduntur enim tunc phaenomena, quae inflammationem in genere denotare solent, plurimum imperfecte evoluta, cum symptomatibus nervosis hyperaesthesiam, vel spasmum indicantibus’ [53]. Another early instance is to be found in Ernst von Grossi’s *Familiarum morborum humanorum expositio*: ‘inflammatio medullae nervae diversae seu neuromyelitis’ [54].

The presence of a *vagina nervorum* as opposed to the medulla was already recognized by earlier authors (who, though, did not use the term ‘nevromyelitis’) as reviewed by Georg Prohaschka in his 1779 *De Structura Nervorum: Tractatus Anatomicus Tabulis Aeneis Illustratus* (see Section I, caput V). However, these early authors (as well as some of the later ones, e.g. Joseph Hyrtl, *Lehrbuch der Anatomie des Menschen*, 1857: ‘Das Neurilemm enthält die Ernährungsgefässe des Nerven, und führt sie seinen Bündelabtheilungen zu’) rather had in mind the connective tissue (epi-, peri-, and endoneurium) investing the nerves, nerve fascicles, and nerve fibres. It was not until 1838/39 that Schwann identified the cell type that commemorates his name, and Ranvier would still bemoan the lack of knowledge and confusion in the nomenclature regarding the nerve sheaths in 1872.

This distinction was soon criticized by Pierre Adolphe Piorry (1794–1879), one of the fathers of percussion and inventor of the plessimeter, as artificial and lacking experimental evidence: ‘A plus forte raison en est-il ainsi du diagnostic qu’on a voulu établir entre la *névromyélite* et la *névrilemmite* [italics ours]. (…) [I]l aurait bien mieux valu la répéter, et, jusqu’à temps qu’on fasse voir le névrilemme d’un filet nerveux enflammé indépendamment de sa pulpe, et vice versa, il sera permis de ne pas croire à ces distinction subtiles qui, à coup sûr, ne sont pas nées d’observations cliniques rigoureuses’ [55]. In 1850, Piorry would repeat his harsh criticism, arguing that the difference would be of little therapeutic consequence: ‘C’est une chose curieuse que de voir l’assurance avec la quelle Boisseau et d’autres pathologistes établissent des caractères distinctifs de la *névrilèmite* et de la névrite qu’ils appellent aussi *névromyélite*. (…) au point de vue pratique a bien peu d’importance. Nous ne voyons pas en effet, quelles seraient les différences à établir sous le rapport du traitement entre la phlegmasie d’un nerf et celle de son enveloppe’ [56].

Piorry’s article was published in 1833 in his *Clinique médicale de l’hôpital de la Pitié et de l’hospice de la Salpétrière*[55] and reprinted in the *Gazette médicale*[57]. English translations appeared soon after in the *American Journal of the Medical Sciences*[58] and in *The Western Journal of the Medical and Physical Sciences*[59]; these translations represent the first uses of the term ‘neuromyelitis’ (N.B.: not ‘neuromyelitis optica’!) we could trace in the English-language literature. Before Piorry, François Gabriel Boisseau (1791–1836) had made the distinction in nomenclature between ‘névrilemmite’ and ’névromyélite’ already in 1830 in his *Nosographie organique*[60], which we believe is the first French account: ‘Quand le névrilème seul est enflammé (*névrilemmite*), (…) Quand la substance médullaire du nerf est elle-même enflammée (*névromyélite*)’ (see also Pierre Joseph Mongellat, *Monographie des irritations intermittentes*, Bruxelles, 1839: ‘isoler l’inflammation du nerf (névrite), de celle du névrilème (névrilémite ou névrilite), et de celle encore de la pulpe nerveuse (névromyélite)’, with reference to Boisseau).

An anonymous review of Hildenbrand’s *Institutiones* in the *Medicinisches Jahrbuch des kaiserlichen königlichen Österreichischen Staates* for 1837 (‘Entzündung (…) der Nervenhäute, *nevrolemitis*, *nevrymenitis*, der Nervensubstanz *nevromyelitis*, *gangliitis*’) is the first account in the German-language literature [61].

Hildenbrand, who coined the term ‘neuromyelitis’, may still be known to some as the eponym of Hildenbrand’s disease, that is typhus (see the back references to ICD-9-CM 081.9). Coincidentally, of all diseases it was Hildenbrand’s disease from which Peppo Acchioté, originator of the term ‘Devic’s disease’, was to die in 1916 [23].

One might accuse us of having overlooked that Piorry referred to Johann Christian Reil (1759–1813) when criticizing the above-mentioned distinction, not to Hildenbrand. Reil, physician to Goethe and appreciated by many as the father of modern psychiatry, was among the first to point to the possibility of inflammation of the nerves by the demonstration of *vasa nervorum* in his seminal *Exercitationum Anatomicarum Fasciculus Primus* / *De Structura Nervorum*, Halle, 1796. Reil also provided a very detailed description of the anatomy of the nerve and its sheaths. In the fourth volume of his main work *Ueber die Erkenntniß und Kur der Fieber*, Vienna, 1802, which is dedicated to feverish nervous diseases, he indeed distinguished afflictions of the *Neurilem* from those of the nervous *Mark*. However, Piorry’s comment leads us astray. A meticulous search of both Reil’s German and Latin writings failed to locate a single mention of the term in question. It is therefore likely that Piorry, when referring to Reil’s *concept*, used the *terminology* of his own time. - Like Acchioté, Reil too died from Hildenbrand’s disease.

### Thomas Clifford Allbutt

Thomas Clifford Allbutt (1836–1925) is still known to many as the inventor of the clinical thermometer and to neurologists and ophthalmologists as the main instigator (alongside Gowers) of the clinical use of the ophthalmoscope. In his famous lecture *On the Ophthalmoscopic Signs of Spinal Disease*[62], Allbutt very briefly mentioned a patient with acute myelitis and ‘a sympathetic eye disorder’. This case was long considered the first account of a patient with NMO in the literature. We recently re-discovered a more detailed description of that patient tucked away in the appendix of Allbutt’s textbook on the use of the ophthalmoscope [17,63]. While Allbutt might not have been the first to report on a case of NMO (see above and reference [17]), it was certainly his report that created the sustained interest of neurologists and ophthalmologists in this rare syndrome.

Often, the palimpsestic syndrome mentioned above is caused by what has been called the Matthew effect: ‘unto every one that hath shall be given’ (Matthew 25:29, King James Version). The incorrect (as we know today) attribution of primacy in relating a case of NMO to Allbutt in so many articles in the field might - given that author’s exceptional reputation and social status (the list of titles held by Allbutt included K.C.B., M.A., M.D., LL.D., D.Sc., F.R.C.P., F.R.S., F.L.S., F.S.A. and Regius Professor of Physic at the University of Cambridge) - indeed represent a true example of that effect. ‘Devic’s disease’, by contrast, is the exception that proves the rule: While Devic became an eponym, pioneers of modern neurology or ophthalmology who were probably better known than Devic at that time and had published on NMO before [11,14], such as Clarke, Allbutt, Erb, or Knapp, came out empty-handed in this case.

Allbutt never used the term ‘neuromyelitis optica’. As a curious coincidence, however, of all authors Allbutt might have been the first to use the term ‘neuromyelitis’ in a medical text in a way distinct from Hildenbrand’s definition. He did so in an address entitled *On the Surgical Aids to Medicine* delivered at the inaugural meeting of the Midland Medical Society on 19th October 1881 [64], in which he referred to the discussion on surgical ‘nerve stretching’ that had taken place at the *International Medical Congress* in August the same year (for the rationale of that unusual procedure see Allbutt’s *System of Medicine* (1901): ‘When all else fails, nerve stretching or even excision of a portion of the nerve must be tried. The amount of tension brought to bear on the nerve should be sufficient at least to lift the limb off the table. One advantage is the breaking down of adhesions, which in a small percentage of cases may have something to do with the pain; but the main advantage is counter-irritation very directly applied.’; cf. also John Marshall, *Neurectasy*, *or*, *Nerve*-*stretching for the relief or cure of pain*: *being the Bradshaw Lecture delivered at the Royal College of Surgeons on the 6th of December 1883*, *with an appendix*, Smith, Elder, and Co, London, 1887). In that address Allbutt concluded that ‘nerve stretching (…) is the only remedy which offers much hope of relief to sufferers from the pains of chronic *neuro*-*myelitis*’. Although Allbutt did not provide an exact definition, he explicitly referred to patients with pain and ‘*spinal* disease’ [italics ours].

### ‘Ascending neuromyelitis’

For the sake of completeness, it should not go unmentioned that the term ‘neuromyelitis’ was used by some authors to refer to Landry’s paralysis, and the World Health Organization’s International Classification of Diseases still lists the term ‘ascending neuromyelitis’ among the synonyms of ‘Guillain-Barre syndrome’ (ICD 61.0). A very rare variant is ‘neuromyelitis hyperalbumenotica’ [65].

### ‘NMO’ in the 20th century: the same term, but evolving concepts

The past 120 years have seen many different criteria for the diagnosis of NMO, and, consequently, the exact meaning (and, in particular, the extension) of the term ‘NMO’ has changed over the years. Some criteria excluded (unnecessarily, as we know today) patients with a relapsing course [66-68], a long interval between the two index events myelitis and neuritis [66-68], only mild para- or tetraparesis [69], incomplete myelitis [69], or unilateral optic neuritis [66-68]. Although Devic and Gault had already pointed to the fact that some patients may develop symptomatic brainstem lesions, some criteria also excluded patients with symptoms other than optic neuritis and myelitis [2,66,70,71] (more recent studies have proven Devic and Gault right [1,9]). Others proposed excluding patients with co-existing systemic lupus erythematosus (SLE) or Sjögren’s syndrome (SS) [70]. However, AQP4-Ab-positive NMO was recently shown to be frequently associated with other autoimmune disorders, in particular SLE and SS [1,72-77]. Finally, some criteria considered spinal cord lesions extending over more than two vertebral segments (as measured by MRI) to be a prerequisite for a diagnosis of NMO [70]. However, recent studies showed that short lesions occasionally occur [1,78]. Similarly, brain lesions on MRI were considered atypical for NMO in the past; later studies, however, demonstrated that (mostly asymptomatic) brain lesions indeed occur in around 60% of patients with NMO, are sometimes present already at onset, and may occasionally even meet MRI criteria for MS [1,79,80].

### NMO and MS: a difficult relationship

The question of the exact relationship between NMO and classical multiple sclerosis (MS) has engaged neurologists for more than a century. While Devic and Gault believed that, ‘des raisons suffisantes pour empêcher d’admettre qu’à aucun moment des lésions aient pu jamais prendre l’apparence anatomique de la sclérose en plaques’ [7], others considered NMO a subtype of MS. For example, Russell Brain, in his famous 1930 review on MS and NMO, concluded that ‘the clinical and pathological differences between neuromyelitis optica and disseminated sclerosis appear to be differences of acuteness and intensity only (…) there seems no justification for separating them’ [81]. Only the discovery of pathogenic antibodies to aquaporin-4 in a subset of patients with NMO but not MS and the demonstration of corresponding pathological differences [4,82-87] has led to the recognition of ‘NMO-IgG-positive NMO’ as an immunopathologically defined disease entity in its own right distinct from classical MS.

All those restrictions in the definition of what the term ‘NMO’ should signify discussed above rested on the need to differentiate NMO and MS solely on the basis of clinical and radiological findings. With the availability of AQP4-IgG, it became possible to distinguish the two conditions on the basis of laboratory findings. This resulted in a change of perspective, which for the first time permitted appreciation of the partial overlap between NMO and MS in terms of clinicoradiological presentation and, in consequence, broadened substantially the meaning attached to the term ‘NMO’.

While clinical and radiological features remain highly relevant when it comes to distinguishing *seronegative* NMO from MS (unless a specific laboratory marker for either of these two conditions is found), the broadening spectrum of syndromes reported associated with AQP4-IgG renders it likely that future diagnostic criteria for *seropositive* NMO will put less emphasis on clinicoradiological findings but rather on strict laboratory standards (this could include the requirement to confirm test results in a second – and, if discrepant, a third –, methodologically independent immunoassay with high specificity and sensitivity as already recommended in current guidelines for other autoantibody mediated diseases of the CNS).

### NMO: disease or syndrome?

The recent progress in our understanding of the pathogenesis of NMO brought about by the breakthrough discovery of AQP4-Ab has challenged the traditional usage of the term ‘NMO’.

Neglecting the large number of reports on cases of possible rheumatic, (para)infectious, paraneoplastic, metabolic, or toxic aetiology, some of which even date back to the time of Devic and Gault, NMO was for a long time treated by many as a disease entity rather than a syndrome, as reflected by the widely applied term ‘Devic’s disease’ (or ‘Morbus Devic’, a variant used mainly, but not exclusively, in the German-language medical literature).

However, the lack of AQP4-IgG-seropositivity in a subset of patients even in the most up-to-date, recombinant assays, and in particular the demonstration of such a lack in some patients with NMO and conditions that may cause myelitis and optic neuritis by other mechanisms, e.g. connective tissue disorders [72], paraneoplastic disorders [88,89], or infectious diseases [90], has provided strong evidence in favour of the hypothesis of NMO being aetiopathogenetically heterogeneous. Importantly, some of these assays have been shown to be capable of detecting AQP4-Ab even in samples taken during remission and under treatment with strong immunosuppressants such as rituximab, azathioprine, mitoxantrone, or cyclophosphamide, practically ruling out the possibility that seronegativity is generally the result of insufficient assay sensitivity [1,91-93]). The notion of aetiopathological heterogeneity is further supported by the recent demonstration of significant clinical and paraclinical differences between seropositive and seronegative patients [1] and the finding of antibodies to myelin oligodendrocyte glycoprotein in some AQP4-Ab-negative patients [94-96].

Nonetheless, the current diagnostic criteria still subsume AQP4-IgG-seropositive and –AQP4-IgG-seronegative cases under the same disease heading, i.e. ‘NMO’ [78]. Employing common criteria and a common designation for seropositive and seronegative NMO may be useful when it comes to differentiating NMO from MS (the lack of oligoclonal bands in most patients with seronegative NMO indicates that the latter is not simply a clinicoradiological subtype of MS but is of distinct pathogenesis [1]); however, this might be problematic when it comes to treating patients with NMO: While the demonstration of a pathogenic effect of AQP4-IgG provides a strong rationale for B cell- and antibody-targeted treatments in AQP4-IgG-seropositive NMO [97,98], so far there is less strong evidence of a role for B cells and pathogenic autoantibodies in the majority of patients with seronegative NMO.

Based on the current concept of NMO being aetiologically heterogeneous (and unless this concept is formally disproved, which is unlikely to happen), the term ‘Devic’s disease’ should be avoided and replaced by ‘Devic’s syndrome’. Accordingly, we believe that the term ‘NMO’ should no longer be used as a *disease* designation; instead, it should be used exclusively to refer to a *clinical phenotype or syndrome* (characterized by optic neuritis and myelitis).

### ‘NMO spectrum disorder(s)’

Another problem attached to the current nomenclature results from the recent finding that the spectrum of clinical manifestations of AQP4 autoimmunity is wider than previously thought and includes (1) various forms of brainstem encephalitis in adults; (2) a broad variety of cerebral symptoms in children; and (3) abortive forms such as isolated longitudinally extensive transverse myelitis or isolated optic neuritis, which in NMO-IgG-positive patients often convert to NMO [1,9,92,99-104]. Some authors proposed tackling this problem by introducing the term ‘NMO spectrum disorder’ (‘NMO-SD’), a designation which was intended to be used to refer to all major clinical manifestations that had by then been reported in association with NMO-IgG.

Unfortunately, however, this potentially useful concept [103] has been employed inconsistently in the literature:

1) Some authors use ‘NMO-SD’ as an abbreviation for ‘NMO spectrum disorder’ (note that ‘disorder’ is used in the singular here) and thereby refer to a shared underlying pathogenesis; by contrast, others defined ‘NMO-SD’ as ‘NMO spectrum disorders’ (note the plural) and thereby refer to a spectrum of clinico-radiological manifestations

2) Among those who understand ‘NMO-SD’ as a spectrum of clinico-radiological manifestations, some include NMO, while others explicitly use the term ‘NMO-SD’ exclusively to refer to manifestations other than NMO

3) Finally, some authors refer only to NMO-IgG-positive cases, while others apply the term both to NMO-IgG-positive and to NMO-IgG-negative cases

A more stringent use of the term ‘NMO-SD’ would be desirable in order to make it easier to compare results between studies. Moreover, it should be taken into account that while most patients with NMO-IgG-positive myelitis, optic neuritis or brainstem encephalitis later convert to NMO, most NMO-IgG-negative patients do not [99,101,104]. Accordingly, labelling all such patients with the term ‘*NMO*-SD’ is somewhat problematic.

### ‘Autoimmune AQP4 channelopathy’

As alternative designations, recently terms based on immunopathology such as ‘autoimmune AQP4 channelopathy’ or ‘autoimmune AQP4 disease’ have been proposed [106-108]. We consider these suggestions useful. These terms could be employed whenever reference is made to the common pathogenenesis thought to underlie the AQP4-IgG-positive cases. We would like to add the term ‘AQP4(−Ab-associated) encephalomyelitis’ as another proposal. This term would be in line with an already established nomenclature that classifies cases of autoimmune encephal(omyel)itis according to the patients’ autoantibody status (e.g. NMDAR encephalitis, anti-Hu-associated encephalomyelitis); moreover, including the word ‘encephalitis’ would take into account (1) that the brain is more often affected than previously thought and (2) that the optic nerve is anatomically part of the encephalon.

### Towards a descriptive and systematic nomenclature

However, for clinical purposes, as well as in the context of clinical studies and treatment trials, neither a classification solely based on common pathogenesis (“AQP4 channelopathy/encephalomyelitis”) nor the (ambiguous) concept of NMO-SD seems completely sufficient; rather, a more differentiated, descriptive and systematic classification that reflects an individual patient’s exact clinical phenotype, antibody status, and disease course might be required, for the following reasons:

First, certain clinical features, laboratory findings, and/or prognostic characteristics differ between AQP4-IgG-positive and AQP4-IgG-negative patients [1,99,101,104,109], among the various NMO-SD (as shown for time-to-relapse, prognostic implications of clinical presentation at onset, and CSF findings [1,110]), and between patients with monophasic disease and patients with relapsing disease [1,2].

Second, treatment strategies might differ between AQP4-IgG-positive and AQP4-IgG-negative patients (taking into account possible differences in aetiology and pathogenesis), even among NMO-SD (e.g. based on differences as to time-to-relapse and short-term prognosis [1]), and between patients with monophasic and patients with relapsing NMO-SD (which could well be manifestations of different diseases, e.g. postinfectious ADEM *vs.* AQP4 autoimmunity).

Third, recommendations on diagnostic AQP4-IgG testing may differ between patients with monophasic and patients with relapsing disease as well as between the various NMO-SDs: given the low frequency of AQP4-IgG in patients with a first attack of isolated optic neuritis [97,98] or atypical, isolated brainstem manifestations on the one hand and the limited specificity of some of the currently available immunoassays on the other hand, general screening for AQP4-IgG in all NMO-SD patients entails the risk of an unfavourably high ratio of false-positive to true-positive results and may therefore not be advisable.

Such descriptive nomenclature would distinguish between AQP4-Ab-positive NMO and AQP4-Ab-negative NMO, AQP4-Ab-positive and AQP4-Ab-negative (longitudinally extensive) myelitis, AQP4-Ab-positive and AQP4-Ab-negative optic neuritis, AQP4-Ab-positive and AQP4-Ab-negative brainstem encephalitis, etc. In addition, the disease course (monophasic/first attack, relapsing) should be specified and information should be provided regarding co-existing autoimmunity as well as regarding the suspected aetiology in seronegative patients.

### Conclusion and outlook

The nomenclature of NMO and its atypical and abortive forms is complex. Its meandering evolution and this complexity reflect the ongoing endeavours by generations of neurologists to facilitate distinction of this rare condition from MS and other related conditions, all the while making room for improvements in diagnosis and in our understanding of the pathogenesis of NMO.

Clear classifications are not a pure academic exercise but a crucial prerequisite for future treatment trials. Our paper aims to fuel the ongoing discussion about the need for new diagnostic criteria for NMO and revisions in nomenclature. We hope that the proposals set out herein will help to solve some of the issues attached to the current nomenclature.

## Abbreviations

NMDAR: N-Methyl-D-aspartate receptor; NMO-SD: Neuromyelitis optica spectrum disorder(s); SLE: systemic lupus erythematosus; SS: Sjögren’s syndrome; ADEM: Acute disseminated encephalomyelitis; AQP4-Ab: Aquaporin-4 antibodies; CNS: Central nervous system; CSF: Cerebrospinal fluid; IgG: Immunoglobulin G; LETM: Longitudinally extensive transverse myelitis; MRI: Magnetic resonance imaging; MS: Multiple sclerosis; NMO: Neuromyelitis optica

## Competing interests

The authors declare that they have no competing interests.

## Authors’ contributions

SJ conceived and designed the study, collected and analysed the data, and drafted the manuscript. BW was involved in revising the manuscript for important intellectual content. Both authors read and approved the final manuscript.
